# Lack of Genetic Differentiation between Contrasted Overwintering Strategies of a Major Pest Predator *Episyrphus balteatus* (Diptera: Syrphidae): Implications for Biocontrol

**DOI:** 10.1371/journal.pone.0072997

**Published:** 2013-09-02

**Authors:** Lucie Raymond, Manuel Plantegenest, Bertrand Gauffre, Jean-Pierre Sarthou, Aude Vialatte

**Affiliations:** 1 INRA, UMR 1201 DYNAFOR, Castanet Tolosan, France; 2 Agrocampus Ouest, UMR 1349 IGEPP, Rennes, France; 3 INRA, USC 1339 (CEBC-CNRS), Beauvoir sur Niort, France; 4 CEBC-CNRS (UPR 1934), Beauvoir sur Niort, France; 5 Université de Toulouse, INPT-ENSAT, UMR 1248 AGIR, Castanet-Tolosan, France; 6 Université de Toulouse, INPT-ENSAT, UMR 1201 DYNAFOR, Castanet-Tolosan, France; U. Kentucky, United States of America

## Abstract

Winter ecology of natural enemies has a great influence on the level and efficiency of biological control at spring. The hoverfly *Episyrphus balteatus* (DeGeer) (Diptera: Syrphidae) is one of the most important natural predators of crop aphids in Europe. Three different overwintering strategies coexist in this species which makes it a good model in order to study ecologically-based speciation processes. The purpose of this study was to determine whether *E. balteatus* populations with alternative overwintering strategies are genetically differentiated. To that aim, we developed 12 specific microsatellite markers and evaluated the level of neutral genetic differentiation between *E. balteatus* field populations that overwinter in the three different ways described in this species (i.e. migration, local overwintering at a pre-imaginal stage, and local overwintering at adult stage). Results showed a lack of neutral genetic differentiation between individuals with different overwintering strategies although there are strong ecological differences between them. All pair-wise F_ST_ values are below 0.025 and non-significant, and Bayesian clustering showed K = 1 was the most likely number of genetic clusters throughout our sample. The three overwintering strategies form one unique panmictic population. This suggests that all the individuals may have genetic material for the expression of different overwintering phenotypes, and that their commitment in one particular overwintering strategy may depend on environmental and individual factors. Consequently, the prevalence of the different overwintering strategies would be potentially modified by landscape engineering and habitat management which could have major implications for biological control.

## Introduction

Insect body temperature varies in relation with the environmental temperature. This results in insect life cycles being highly dependent on climatic conditions. Therefore, in temperate regions, adaptation to winter conditions is an important life history trait that may influence the ecological and evolutionary success of insects. To cope with adverse winter conditions, insects have developed a great variety of ecological strategies including migration [1], [2] and diapause [3] that can occur simultaneously in the same species [4]. Alternative overwintering strategies may lead to allopatry or allochrony in reproduction between individuals adopting distinct strategies, resulting in a reduction of genetic mixing. Migration is notably known in birds to be involved in genetic divergence between sedentary and migratory populations, or between populations displaying different migration patterns [5], [6].

Various processes may lead to reproductive isolation and genetic divergence between populations adopting distinct overwintering strategy. First, differences in overwintering strategy can lead to temporal segregation in adults spring appearance and consequently to assortative mating within each strategy [7]. This allochronic isolation – separation of populations by breeding time - may lead to population divergence and speciation: *Oceanodroma castro* (Harcourt) (the Madeiran storm-petrel) exhibits very reduced or even completely disrupted gene flow between populations that breed in the same places but in different seasons [8]. Moreover, overwintering strategy shapes a part of the fitness of individuals and is potentially under strong selection [9]. This selection, if existing, may cause indirect selection on genetically correlated traits that could have consequences beyond the winter period. For example, it is well established that a significant part of migratory phenotype has genetic bases and has necessarily some correlation with other traits owing to pleiotropy or linkage [10]. These correlated traits might affect breeding preferences and their indirect divergent selection might lead to reproductive isolation between migratory and non-migratory individuals. Genetic isolation and population differentiation based on different overwintering phenotypes has been the subject of many studies in birds, but these processes have been little investigated in insects until now.

The hoverfly *Episyrphus balteatus* (DeGeer) (Diptera: Syrphidae), at its larval stage, is one of the most important aphid feeding predators in Europe [11]–[13]. During the primary phase of an aphid infestation in cereal fields, it may significantly reduce the population growth rate of the pest [13]. Thus, hoverflies that are present and active in early spring could allow keeping aphid populations below damaging levels. It is very likely that the precocity of field colonization by hoverflies largely depends on the overwintering strategy during the previous winter. Consequently, identifying internal or ecological factors determining the commitment to an overwintering strategy in this species is crucial to determine conditions favouring biological control of aphid in agricultural fields and to enhance biological control through ecological engineering. Three different overwintering strategies have been described for *E. balteatus*. Some individuals overwinter as adults in a facultative reproductive diapause [14], [15] mainly in south facing edge habitats providing both shelter and nutritional resources [16]. Others overwinter at a pre-imaginal stage in the soil or litter of field boundaries [16], [17]. Finally, some individuals perform long distance migration southwards during autumn [18]. The coexistence of three different strategies in the species makes it a good model to study the impact of overwintering strategies on reproductive isolation in insects. However, the winter ecology in this species has never been considered from an evolutionary point of view to date, and we still do not know if there is genetic divergence between individuals overwintering with different strategies.

The purpose of this study was to assess whether *E. balteatus* adopting alternative overwintering strategies are genetically differentiated. To achieve this objective, 12 *E. balteatus* specific microsatellite markers were developed and specific sampling protocols were adopted to collect individuals belonging to the three overwintering strategies in four different study sites. Because of allochrony and different environmental pressures, we expected to observe a genetic differentiation between individuals adopting alternative overwintering strategies. Finally, we found no genetic divergence between the different strategies and sampling sites, which may have several implications for aphid biological control.

## Materials and Methods

### Study Sites and Insect Sampling

**Figure 1:**
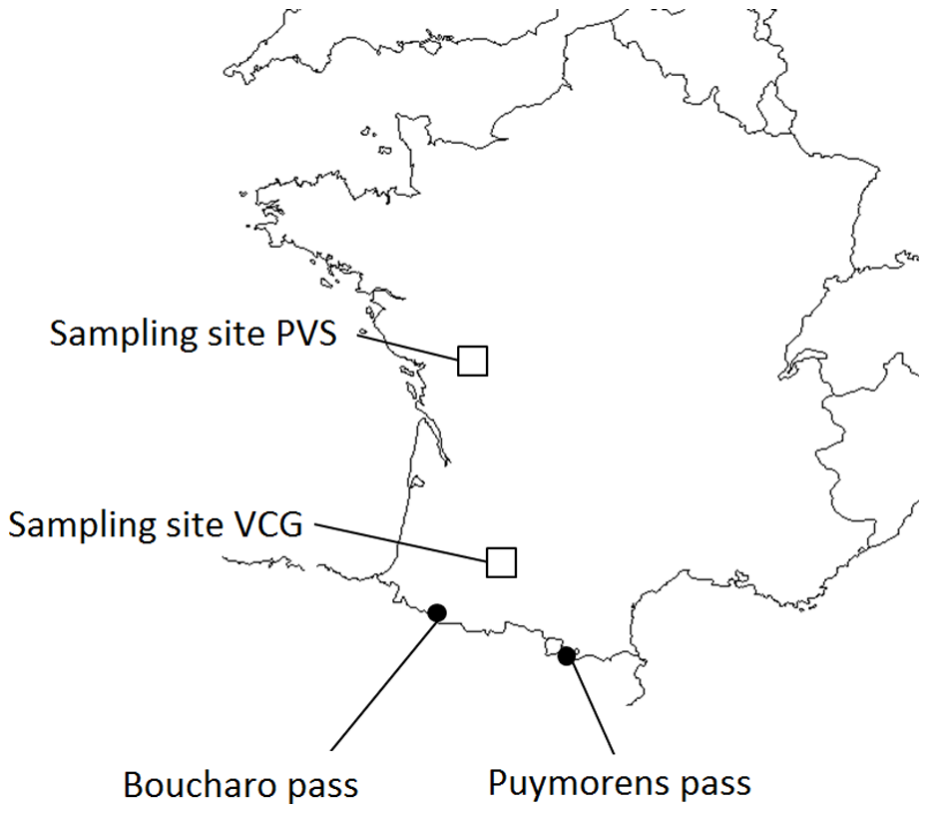
Sampling sites locations. Sampling sites VCG ("Vallées et Coteaux de Gascogne"; 43°17′ N, 0°54′E ) and PVS (“Plaine et Val de Sèvre”; 46°2′N,0°4′W) were sampling sites for the overwintering strategies at adult and pre-imaginal stages, Boucharo pass (42°42′13″N, 0°3′52″O) and Puymorens pass (42°33′35″N, 1°48′37″E) were sampling sites for migratory individuals.

We used *E. balteatus* field populations that overwinter through different strategies. Individuals overwintering locally at a pre-imaginal or adult stage were collected during two years (2011 and 2012) in two French sites distant from each other 400 km (Fig. 1). The study site “Vallées et Coteaux de Gascogne” (VCG) is a 220 km^2^ hilly area located in south-western France (43°17′ N, 0°54′ E) and is part of the Long Term Ecological Research network (LTER_EU_FR_003). The study site “Plaine et Val de Sèvre” (PVS) is located in west France (46°2′N, 0°4′W) and covers a 450 km^2^ area of intensive agriculture. The choice of the sampling protocol allowed distinguishing unambiguously populations according to their overwintering strategy. Individuals overwintering as adults were caught by Malaise traps (surface: 1.8 m^2^; B&S Entomological Services, Co. Armagh, N. Ireland, UK) during the winters 2011 and 2012. Traps were preferentially placed along south facing forest edges or along south facing hedges. Twelve Malaise traps were used in the VCG study site between 12-Jan-2011 and 08-Mar-2011 and ten between 22-Dec-2011 and 16-Mar-2012. Ten Malaise traps were used in the PVS study site between 22-Dec-2011 and 16-Mar-2012. Individuals overwintering at a pre-imaginal stage were caught in 2011 with emergence traps between the end of winter and the beginning of summer which is the period of adult emergence [17]. Traps were placed at the two study sites in cereals, oilseed rape, and alfalfa fields as well as at adjacent grassy boundaries adjoined or not by hedges. They were installed on the 20-Mar-2011 and specimens were caught from this date until the 01-Jul-2011. We used 60 small size traps (surface: 0.36 m^2^, Soil Emergence trap 96×26 mesh, Black, MegaView Science Co., Ltd, Taichung, Taiwan) in the PVS study site, and 30 large traps (surface: 1.8 m^2^, a modified Malaise trap to the design of M.C.D. Speight; B&S Entomological Services, Co. Armagh, N. Ireland, UK) in the VCG study site. The use of different size emergence trap was due to material availability in the two study sites. Collecting bottles of Malaise traps and emergence traps were filled two thirds full with 70° ethanol. In addition, migratory individuals were caught in the Pyrenees, on their migratory route towards Southern Europe. Two mountain passes (Boucharo pass, 42°42′13″N, 0°3′52″O, altitude 2273 m; and Puymorens pass, 42°33′35″N, 1°48′37″E, altitude 1920 m) were chosen as sampling sites as they have been previously described as migration passes [19], and because they do not represent suitable habitats for *E. balteatus* due to their high altitude. Migratory individuals were caught between 01-Sep-2011 and 02-Oct-2011 using an interception trap with a 4 m triangular opening described in Aubert [20]. All specimens were manually sorted out and identified, and *E. balteatus* individuals were stored individually in Eppendorf tubes in 90° ethanol, and kept at 4°C prior to molecular analyses.

The emergence traps and Malaise traps were installed on private lands whose owners had given permission to conduct the study on these sites. Authorizations for sampling at Boucharo pass and Puymorens pass were respectively obtained from the Parc National de Pyrénées and from the Parc Naturel Régional des Pyrénées Catalanes. Sampling for this study did not involve endangered or protected species.

We defined six groups on the basis of the overwintering strategy and the sampling site: overwintering pre-imaginal individuals caught in site VCG (P_VCG_), overwintering pre-imaginal individuals caught in site PVS (P_PVS_), overwintering adults caught in site VCG (A_VCG_), overwintering adults caught in site PVS (A_PVS_), migratory individuals caught at Boucharo pass (M_Bou_), migratory individuals caught at Puymorens pass (M_Puy_). We also aggregated the groups from different sampling sites sharing the same overwintering strategy (i.e. individuals overwintering locally at pre-imaginal stage (P), individuals overwintering locally at adult stage (A), individuals overwintering by migrating (M)) in order to test for genetic differentiation between overwintering strategies independently from sampling site.

### DNA Extraction and Microsatellite Amplification

Genomic DNA was extracted from insect head and thorax by a “salting out” protocol (method in Sunnucks and Hales [21]). The DNA was then re-suspended in 250 mL of ultra-pure water and stored at −18°C. Genotypes of *E. balteatus* specimens were obtained for 12 *E. balteatus* specific nuclear microsatellite markers newly developed from a commercially made microsatellite-enriched library (Genoscreen; Lille, France) (Table 1). Microsatellite amplification and genotyping were carried out at the GENTYANE genomic platform (Clermont-Ferrand, France). Fragment amplification was done in a 10 µL polymerase chain reaction (PCR) reaction volume, using 5 µL AmpliTaq Gold® 360 Master Mix (AB-life technologies). Each reaction contained 20–50 ng DNA templates and 0.5 µM of each primer. Forward primers were labelled with fluorescent dyes. Amplifications were made in a thermocycler Veriti® 384 Well (AB-life technologies). The cycling profiles consisted of 10 min initial denaturation at 95°C, followed by 7 cycles of 30 s at 95°C, 30 s at 62°C (−1°C/cycle) and 30 s elongation at 72°C, 30 cycles of 30 s at 95°C, 30 s at 55°C and 30 s at 72°C, 8 cycles of 30 s at 95°C, 30 s at 56°C and 30 s at 72°C, and 5 min at 72°C to end the reactions. The reactions were then chilled at 15°C. Fragments were separated on 3730xl DNA Analyser (AB-life technologies), and microsatellite allele sizes were scored using the software GeneMapper® 4.0 (AB-life technologies).

**Table 1:** Characteristics of the microsatellite markers used in the study.

Marker name	Primer sequence (5′-3′)	N_A_	A_S_	Repeatmotif	Missing data (%)		GENEBANK accession number	
Ba13	**Ba13F** CTTTACACTCTTACGCGCCC **Ba13R** TGAGAAGACGACACAGCGTT	14	102–146	gtt		0		KF419302
Ba23	**Ba23F** ATTTTTGTGGACATTAAAGTGATTT **Ba23R** GCTAAAAGGGTGTTTGGGGT	8	149–169	tg		0.3		KF419303
Ba25	**Ba25F** AACAACTTTCGTCGGGTTTC **Ba25R** TCACGCCTGAAACACAAAAC	20	143–181	ct		0		KF419304
Ba3	**Ba3F** GACAATTGAACAGTCTGCTGC **Ba3R** TCGAAGAACAAATAAACATCGAA	8	107–121	ct		0.5		KF419305
Ba30	**Ba30F** TGATTTCAATTAATCAGGAAGTCG **Ba30R** TCCAGCGTTACATCAAGGTG	28	169–220	ct		0		KF419306
Ba32	**Ba32F** ATGTACCGCTTGCTTTCGTT **Ba32R** CGACTTGATTGAACTCTGCTG	12	182–218	caa		0		KF419307
Ba33	**Ba33F** TTGTCATCAGTTCGTTTCATCC **Ba33R** GACCACCATCACCACCATTA	13	162–220	aac		0		KF419308
Ba35	**Ba35F** TGGGCACTATTCAACGGAA **Ba35R** CGTTCTTATTTGATGCACCG	17	199–223	tc		0		KF419309
Ba46	**Ba46F** CAAAGGCATCATATCCGATTCT **Ba46R** ATTTCATTTGATTGCGGAGC	12	266–291	ga		0.3		KF419310
Ba7	**Ba7F** CACCAAGTGCAATCGAAGTG **Ba7R** TTATCACACCGTTCGACGC	13	105–130	tg		0.3		KF419311
Ba8	**Ba8F** GAAATCCGGCCATCACATAC **Ba8R** AGGTGCTGCTCTGGTTTGTT	13	116–135	acg		0		KF419312
Ba9	**Ba9F** ACAAATGAATGTTTCATGTCGAT **Ba9R** TCGTTTGAGATATTAAGAGCAACA	27	103–172	ac		0		KF419313

Locus name, primer sequence (F: forward primer, R: reverse primer), number of alleles over the 6 sampling groups (N_A_), allele size range (A_S_), repeat motif, proportion of missing data (%), GENEBANK accession number.

### Microsatellite Data Analyses

First, we checked for the presence of null alleles for each locus and sampling group by calculating the proportion of missing data (Table 1) and testing for homozygote excess in the different alleles with Microchecker 2.2.3 [22]. The software Arlequin 3.5.1.3 [23] was used to calculate observed heterozygosity, expected heterozygosity, and deviation from Hardy-Weinberg equilibrium at each microsatellite locus in each of the six *E. balteatus* sampling groups (P_VCG_, P_PVS_, A_VCG_, P_PVS_, M_Bou_ and M_Puy_). The significance of differences between expected and observed heterozygosities (α = 0.05) was determined using Arlequin 3.5.1.3 after a sequential Bonferonni correction carried out on the 12 statistical tests in each group. We also used Arlequin 3.5.1.3 to calculate linkage disequilibrium between all pairs of loci in the total population and its significance by an exact Fisher test on 20000 permutations (α = 0.05) after sequential Bonferroni correction.

One locus (Ba13) displayed evidences for the presence of null alleles and was thus removed for subsequent analyses.

### Genetic differentiation between Overwintering Strategies and Sampling Groups

The genetic structure and diversity among the six sampling groups and the three overwintering strategies were assessed using genotypes across 11 microsatellite loci. For each sampling group and each overwintering strategy, the mean expected and observed heterozygosity (H_e_ and H_o_) were calculated using Arlequin 3.5.1.3, the mean number of alleles per locus (A_N_), the multilocus inbreeding coefficient (F_IS_), and the allelic richness (R_S_) were calculated using Fstat 2.9.3.2 [24]. The rarefaction procedure used to compute allelic richness gives an unbiased measure of the number of alleles estimated independently of sample size, hence allowing comparing this quantity between different sample sizes [25]. The levels of significance for the F_IS_ were obtained after 1320 randomizations of alleles among individuals within sampling groups and after 660 randomizations of alleles among individuals within overwintering strategies, as implemented in Fstat 2.9.3.2 [24].

The level of neutral genetic differentiation between sampling groups and overwintering strategies was quantified using the F_ST_ [26]. Pair-wise F_ST_ between sampling groups and overwintering strategies, and their significance with 10 000 bootstrap replicates were assessed using Arlequin 3.5.1.3 [23]. A locus by locus analysis of molecular variance (AMOVA) [27] after 10 000 permutations were performed using Arlequin 3.5.1.3 in order to determine the relative contribution of within-sampling groups, between-sampling groups and between overwintering strategies genetic diversity to the overall genetic diversity. The software Powsim 4.1 [28] was used to evaluate with which statistical power our microsatellites and observed allele frequencies would allow determination of significant genetic differentiation between sampling groups and overwintering strategies. We simulated the sampling of an effective population size of 1000 individuals into three or six populations, reflecting the sample sizes of the overwintering strategies and sampling groups used in the study and based on a random drawing of alleles that occurred at the overall frequencies observed in our total sample. Simulations were carried out for a series of seven dictated F_ST_ values between 0 and 0.05 with 1000 runs per F_ST_ value. Statistical power was determined as the proportion of simulations for which Fisher’s exact tests showed a significant deviation from 0 (i.e. significant genetic differentiation) [28].

Finally, population structure was inferred using a Bayesian clustering algorithm implemented in the computer program Structure 2.3.3 [29]. In Structure, the number of clusters (denoted by K hereafter) representing the data was explored by performing 20 replicates of each simulation from K = 1 to K = 9, with a burn-in of 50 000 steps followed by 500 000 Markov chain Monte Carlo (MCMC) iterations under the admixture model and the assumption of correlated allele frequencies among populations. Individuals were assigned to clusters based on their highest membership coefficient to a particular cluster averaged over the twenty independent runs. The K value which better fitted our genetic data was detected using the highest likelihood method [29]. We did not use the Δk method of Evanno [30] because the calculation method of Δk does not allow to provide a value for K = 1.

## Results

### Microsatellite Data Analyses

A total of 376 individuals (sample sizes given in Table 2) were genotyped at 12 microsatellite loci (0.1% missing data). The proportion of missing data per locus ranged between 0 and 0.5%. No test of pair-wise linkage disequilibrium was significant. Several loci showed significant departure from Hardy-Weinberg equilibrium in only one of the six sampling groups (Ba9, Ba23, Ba25, Ba33, Ba35, and Ba46) (Fig. S1). One of the twelve loci (Ba13) showed significant departure from Hardy-Weinberg equilibrium in four of the six sampling groups. The computer program Microchecker 2.2.3 [22] detected the possibility of null alleles in several sampling groups and loci because of excess of homozygotes in most allele sizes (loci Ba13, Ba33, and Ba9 in the sampling group M_PUY,_ and loci Ba13, Ba30, Ba33, Ba35 and Ba9 in the sampling group M_BOU)_. Particularly, the locus Ba13 showed evidence for null alleles in four of the six sampling groups (A_VCG_, A_PVS_, M_PUY_ and M_BOU_). We considered that homozygote excess was due to chance or was an effect of sample size when it was significant only in one sampling group. In this case, we conserve the involved loci for population structure analyses. When a locus displayed homozygote excess in more than three of the six sampling groups, we considered that it could be due to the presence of null allele and removed this locus for subsequent analyses. In this way, the locus Ba13 was discarded. However, we note that the presence of this locus does not influence the results and conclusions of this study.

**Table 2:** Genetic diversity in sampling groups and overwintering strategies.

		N	A_N_	R_s_	H_o_	H_e_	F_IS_
	P_VCG_	15	7.45	4.48	0.74	0.72	0.00 n.s
	P_PVS_	5	4.27	4.27	0.53	0.67	0.23n.s
	A_VCG_	58	10.18	4.32	0.67	0.70	0.05 n.s
Sampling groups	A_PVS_	42	9.73	4.33	0.71	0.71	0.00 n.s
	M_Bou_	197	13.18	4.29	0.66	0.70	0.06 n.s
	M_Puy_	59	10.18	4.28	0.64	0.69	0.07 n.s
	P	20	7.9	7.9	0.69	0.71	0.03 n.s
Overwintering	A	100	11.7	7.4	0.68	0.70	0.03 n.s
strategies	M	256	14.1	7.3	0.65	0.70	0.06 n.s

Number of individuals sampled (N), mean number of allele per locus per sampling group or overwintering strategy (A_N_), mean allelic richness corrected for sample size of 5 individuals for sampling groups and 20 individuals for overwintering strategies (R_S_), expected heterozygosity under HW conditions (H_e_), observed heterozygosity (H_o_), inbreeding coefficient (F_IS_) and its statistical significance (*P<0,05; n.s non-significant).

### Genetic Diversity in Sampling Groups and Overwintering Strategies

We found 171 alleles over the 11 selected loci and the six populations (Table 2). Loci Ba23 and Ba3 showed the lowest number of alleles (8) and locus Ba30 showed the greatest number of alleles (28). The mean number of alleles per locus was 15.5. The allelic richness over the 11 loci was not significantly different between sampling groups (ANOVA, F = 0.04, *P = *0.99) neither between overwintering strategies (ANOVA, F = 0.11, *P* = 0.89). Among sampling groups, the mean observed heterozygosity ranged from 0.53 to 0.74 and the mean expected heterozygosity ranged from 0.67 to 0.74. Among strategies, the mean observed heterozygosity ranged from 0.65 to 0.69 and the mean expected heterozygosity ranged from 0.70 to 0.71. Except for the sampling group P_PVS_ (F_IS = _0.23), multilocus inbreeding coefficient (F_IS_) among sampling groups and overwintering strategies displayed low values (between 0.01 and 0.07). Permutation test revealed that none of F_IS_ value was significant. The high but non-significant F_IS_ value of the sampling group P_PVS_ is probably an effect of the small size of this group (n = 5).

### Genetic Differentiation between Sampling Groups and Overwintering Strategies

Neutral genetic divergence either between sampling groups or between overwintering strategies (Table 3) estimated by F_ST_ was very low. Pair-wise F_ST_ values varied from 0 to 0.022 and none was significant. A global test of differentiation among samples showed no differentiation (exact *P* = 1 on 30 000 Markov chains). The analysis of molecular variance (AMOVA) confirmed a lack of differentiation between sampling groups and between overwintering strategies: more than 99% of the molecular variance was attributed to the within-group variance although less than 1% was attributable to a differentiation between groups within strategies or between strategies (Table 4).

**Table 3:** Pair-wise F-statistics (F_ST_) between sampling groups (a.) and overwintering strategies (b.).

a.		M_Bou_	M_Puy_	A_VCG_	A_PVS_	P_VCG_	P_PVS_
	M_Bou_		n.s	n.s	n.s	n.s	n.s
	M_Puy_	0.002		n.s	n.s	n.s	n.s
	A_VCG_	0.002	0.003		n.s	n.s	n.s
	A_PVS_	0.000	0.001	0.000		n.s	n.s
	P_VCG_	0.000	0.000	0.000	0.000		n.s
	P_PVS_	0.015	0.022	0.005	0.011	0.004	

b.		M	A	P
	M		n.s	n.s
	A	0.001		n.s
	P	0 .000	0.000	

F_ST_ values in the lower matrix and significance in the upper matrix (*P<0.05; n.s. not significant)

**Table 4:** Result of Analysis of Molecular Variance (AMOVA) comparing the six sampling groups and the three overwintering strategies.

Source of variation	d.f	Sum of squares	Variance components	Percentage variation	*P*
Among strategies	2	8.828	0.00195 Va	0.05	0.331
Among groups within strategies	3	12.199	0.00218 Vb	0.06	0.441
Among individuals within groups	46	2874.140	3.85273 Vc	99.89	0.000
Total	51	2895.168	3.85686		

**Figure 2:**
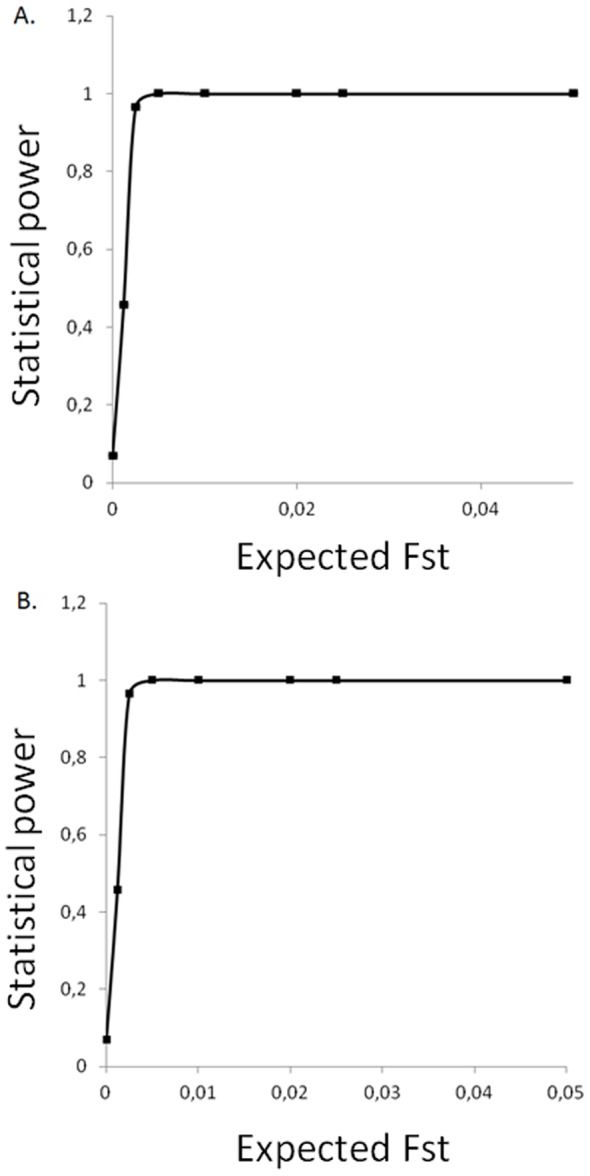
Statistical power in detection of significant genetic differentiation. Statistical power is given as calculated by Powsim 4.1 (Ryman and Palm 2006). A. Simulated sampling of Ne = 1000 into six populations reflecting sample sizes of A_VCG_, A_PVS_, P_VCG_, P_PVS_, M_Bou_, M_Puy_ sampling groups. B. Simulated sampling of Ne = 1000 into three populations reflecting sample sizes of A, P and M overwintering strategies.

The simulations carried out with the software Powsim 4.1 [28] based on the microsatellite data showed that the 11 microsatellite markers used in the population genetic study would be able to detect F_ST_ values as low as 0.0025 in 98% of the cases for the simulated sampling of an effective population size of 1000 into three populations, and in 96% of the cases for the simulated sampling of an effective population size of 1000 into six populations (Fig. 2). These results eliminate the possibility of a type 2 error (false negative) for the population differentiation.

### Population Structure

The Bayesian assignment performed with the software Structure 2.3.3 [29] revealed no genetic structure in our sample, neither based on overwintering strategy nor on geographical origin. K = 1 was the most probable number of genetic clusters to explain our data (estimated ln probability of data = −13499.5, *P*>0. 99) (Fig. S2).

## Discussion

Ecological differences between *E. balteatus* individuals overwintering with different strategies led us to hypothesize reproductive isolation among them. According to the classification proposed by Rundle and Nosil [31], *E. balteatus* winter ecology is likely to produce three forms of reproductive isolation involved in ecological speciation: habitat isolation, temporal isolation and natural selection against immigrants. With (i) larvae or pupae overwintering in soil or litter of cultivated fields and grassy margins, (ii) fertilized females overwintering actively in forest edges and (iii) migrating adults overwintering at few hundred kilometres further south, *E. balteatus* exhibits both divergent habitat and developmental schedules that may induce strong differences between individuals in abiotic conditions and in the suit of predators and competitors they face. These environmental differences are potential ecological causes of divergent selection because of the divergent pressures they induce on individuals [32]. Moreover, like most migratory insects [33], *E. balteatus* probably has a continuously breeding migration pattern, with each individual carrying out only a single part of the migratory circuit. Owing to local adaptation to origin region conditions, this migration pattern may cause differences between individuals coming from southern Europe at spring to colonize middle and northern Europe and individuals overwintering locally in these regions. These differences may induce selection against immigrants which is another potential cause of reproductive isolation.

Ecological speciation was previously shown in relation to differences in spring emergence time in *Rhagoletis pomonella* (Walsh) (the apple maggot fly) [34], habitat in *Dalbulus maidis* (Delong & Wolcott) (a specialist corn leafhopper) [35], or selection against immigrants in walking–stick insects [36]. However, the present results clearly demonstrate that there is no reproductive isolation between populations using different overwintering strategies in *E. balteatus.* Low F_ST_ values between sampling groups and overwintering strategies, extremely low contribution of between-sampling groups and between-overwintering strategies differentiation to the total amount of molecular variance and absence of genetic structure identified by genetic clustering are consistent with the fact that *E. balteatus* individuals belong to a unique panmictic population at large geographic scale and irrespective to their overwintering strategies. The genetic mixing between individuals performing different overwintering strategies results in the absence of genetic differentiation between sampling sites. Indeed, the long range migratory individuals should allow gene flow between distinct geographical zones and act as a genetic homogenization force. Genetic mixing is also facilitated by the multivoltin ecological cycle of *E. balteatus*. The several overlapping generations during the summer period would allow erasing the ecological adaptive differences observed during the winter period.

Although our results demonstrate there is no reproductive isolation between the overwintering strategies, we cannot conclude on the absence of divergent selection on them. Differences in overwintering strategy may result from selection on relatively few genomic regions and is not necessarily picked up by neutral genomic markers, as mentioned by Liedvogel *et al*. [10] for migratory traits. There are some examples of populations under different selection pressures that do not display neutral genetic differentiation [37], [38]. In particular, eastern and western American populations of the monarch butterfly (*Danaus plexippus* L.) are not genetically differentiated on neutral markers despite different migration patterns and distinct overwintering sites [39]. It is known that migration and diapause have genetic bases [3], [10] but the phenotypic variation between individuals adopting such strategies might not necessarily be found in gene sequences responsible for these phenotypes but rather in regulatory elements of gene expression [40]. In *E. balteatus* all individuals may have the genetic material for the expression of all three phenotypes, and their commitment in one particular overwintering strategy may be due to differential gene expression in response to environmental conditions, level of energetic resources, and age. Transcriptomic approaches could allow validating this hypothesis in the future.

Adoption of a particular overwintering strategy in *E. balteatus* can be understood as an ecological opportunity. Given that the females lay eggs continuously during more than one month [41], one possible scheme is that the first eggs laid by females fertilized at the end of the summer would have enough time and resources to reach the adult stage and migrate or stay and overwinter locally depending on the environmental conditions and the level of resources. Eggs laid later by the same females would not be able to reach adult stage and would overwinter locally at pre-imaginal stage. In this scheme the descendants from the same parents could adopt distinct overwintering strategies what is consistent with the lack of genetic differentiation between these strategies. A relatedness analysis would be useful to identify strategies with more full sibs, but the probability to catch full sibs in different strategies with our sampling design is very low, as we catch insects in different places and seasons for each strategy. The investment in several overwintering strategies for a same lineage leads to distribute reproductive effort across a number of events under the uncertainties of the environment in winter. It allows delaying or skipping reproduction in unfavourable conditions and engaging in reproduction when conditions are favourable. This incredible plasticity in *E. balteatus* is certainly a cause of the ecological and evolutionary success of this species which can be found in many different habitats from Palaeartic to Afrotropical, Oriental or Australian regions [42], [43]. It also allows maintaining populations in disturbed habitats such as agricultural fields and contributes to the efficiency of aphid biological control by this species. If the commitment to a particular overwintering strategy effectively responds to combined environmental and individual factors, the implications in population management for biological pest control would be strong. It would mean that it may be possible to influence the prevalence of the different strategies by ecological engineering. For example, we could increase the proportion of the population locally overwintering by providing habitats and trophic resources, in order to reduce the dependence on spring arrival of immigrants for aphid biological control. If most of individuals would overwinter locally, the planning of the biological control would be easier because it would mostly depend on local winter conditions. For example, the model Hover-Winter [44], gives predictions of survival rate and spatial distribution of an *E. balteatus* adult overwintering population from landscape and climatic input data.

This study is the first population genetics study based on microsatellite markers on *E. balteatus* which is a major beneficial insect in agriculture. Our results clearly state the lack of neutral genetic differentiation in this species at a large spatial scale, irrespective to the overwintering strategies of individuals. The lack of genetic differentiation could help improve biological control against aphids, as it would be possible to increase the proportion of the population locally overwintering by landscape engineering. Future studies that could focus on the factors responsible for the commitment to a particular overwintering strategy, based on next-generation sequencing technologies could confirm this hypothesis and determine precisely the necessary conditions for the local overwintering and therefore a more effective biological control.

## Supporting Information

Figure S1 Expected (He) and observed (Ho) heterozygosity at each locus in the six groups. Significance differences between these values (determined with an α of 0.05 and using a sequential Bonferroni correction (Rice 1989)) are indicated by asterisks. Loci for which at least two groups showed significant departure from Hardy–Weinberg equilibrium were discard for subsequent analyses and are indicated in grey shading.
(DOCX)

Figure S2 Bayesian assignment probabilities for k = 2, K = 3 and K = 4. Each vertical line represents an individual, and colours indicate the proportion of an individual’s genotype assigned to a particular lineage, individuals are sorted by overwintering strategy and sampling site.
(DOCX)
