# Supplementary material for: Lack of Genetic Differentiation between Contrasted Overwintering Strategies of a Major Pest Predator Episyrphus balteatus (Diptera: Syrphidae): Implications for Biocontrol
Source: PLoS One. 2013 Sep 2;8(9):e72997. doi: 10.1371/journal.pone.0072997 (PMC3759392; doi:10.1371/journal.pone.0072997)
Supplement: Figure S1 — Expected (He) and observed (Ho) heterozygosity at each locus in the six groups. Significance differences between these values (determined with an α of 0.05 and using a sequential Bonferroni correction (Rice 1989)) are indicated by asterisks. Loci for which at least two groups showed significant departure from Hardy–Weinberg equilibrium were discard for subsequent analyses and are indicated in grey shading. (DOCX) [file pone.0072997.s001.docx]

Figure S1, Supporting Information.

|  | A_VCG_ | | A_PVS_ | | P_VCG_ | | P_PVS_ | | M_Bou_ | | M_Puy_ | |
| --- | --- | --- | --- | --- | --- | --- | --- | --- | --- | --- | --- | --- |
| Locus | Ho | He | Ho | He | Ho | He | Ho | He | Ho | He | Ho | He |
| Ba13 | 0.58 | 0.74* | 0.64 | 0.80* | 0.53 | 0.75 | 0.60 | 0.89 | 0.62 | 0.79* | 0.64 | 0.77* |
| Ba23 | 0.48 | 0.49 | 0.52 | 0.52 | 0.60 | 0.55 | 0.60 | 0.53 | 0.46 | 0.48* | 0.37 | 0.45 |
| Ba25 | 0.74 | 0.76 | 0.74 | 0.74 | 0.93 | 0.84 | 0.60 | 0.67 | 0.78 | 0.82* | 0.80 | 0.83 |
| Ba3 | 0.57 | 0.65 | 0.62 | 0.63 | 0.73 | 0.66 | 0.40 | 0.36 | 0.59 | 0.62 | 0.64 | 0.64 |
| Ba30 | 0.91 | 0.91 | 0.88 | 0.91 | 0.80 | 0.89 | 0.80 | 0.96 | 0.82 | 0.89* | 0.88 | 0.90 |
| Ba32 | 0.76 | 0.70 | 0.76 | 0.72 | 0.67 | 0.67 | 0.20 | 0.56 | 0.67 | 0.70 | 0.63 | 0.66 |
| Ba33 | 0.78 | 0.83 | 0.86 | 0.81 | 0.87 | 0.79 | 0.60 | 0.73 | 0.75 | 0.80 | 0.61 | 0.77* |
| Ba35 | 0.67 | 0.74 | 0.79 | 0.78 | 0.80 | 0.74 | 0.60 | 0.64 | 0.63 | 0.74* | 0.76 | 0.73 |
| Ba46 | 0.43 | 0.51 | 0.55 | 0.51 | 0.53 | 0.45 | 0.40 | 0.53 | 0.41 | 0.45* | 0.44 | 0.42 |
| Ba7 | 0.67 | 0.71 | 0.69 | 0.76 | 0.80 | 0.71 | 0.40 | 0.73 | 0.73 | 0.72 | 0.68 | 0.70 |
| Ba8 | 0.60 | 0.68 | 0.67 | 0.59 | 0.53 | 0.75 | 0.60 | 0.78 | 0.65 | 0.67 | 0.56 | 0.68 |
| Ba9 | 0.71 | 0.76 | 0.71 | 0.80 | 0.87 | 0.83 | 0.60 | 0.84 | 0.75 | 0.81* | 0.71 | 0.82 |
