# Supplementary figures and images for: Lack of Genetic Differentiation between Contrasted Overwintering Strategies of a Major Pest Predator Episyrphus balteatus (Diptera: Syrphidae): Implications for Biocontrol
Source: PLoS One. 2013 Sep 2;8(9):e72997. doi: 10.1371/journal.pone.0072997 (PMC3759392; doi:10.1371/journal.pone.0072997)

Figure S2, Supporting Information


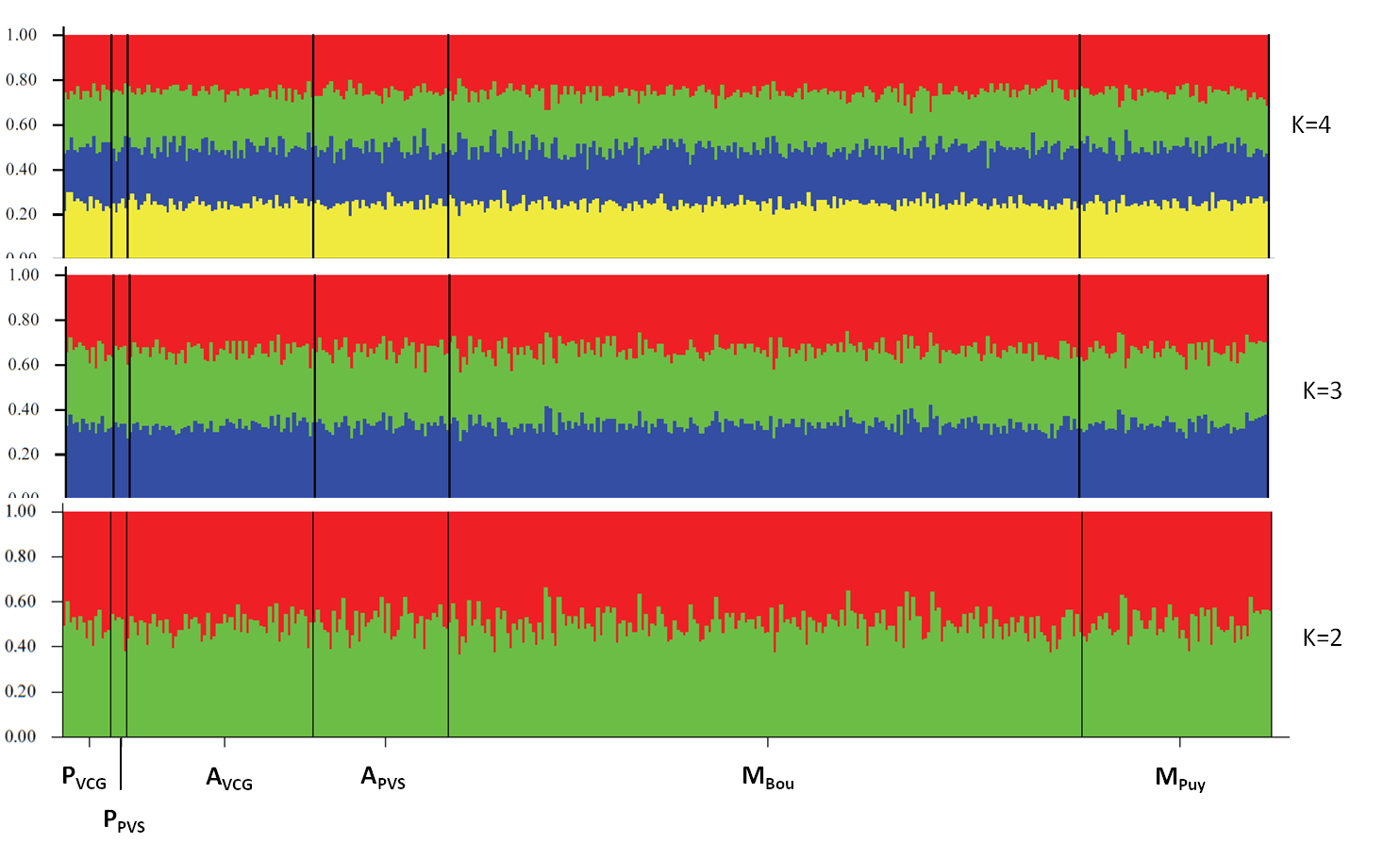

Supplement: Figure S2 — Bayesian assignment probabilities for k = 2, K = 3 and K = 4. Each vertical line represents an individual, and colours indicate the proportion of an individual’s genotype assigned to a particular lineage, individuals are sorted by overwintering strategy and sampling site. (DOCX) [file pone.0072997.s002.docx]
